# *PARK2* loss promotes cancer progression via redox-mediated inactivation of PTEN

**DOI:** 10.1080/23723556.2017.1329692

**Published:** 2017-05-19

**Authors:** Amit Gupta, Sara Anjomani-Virmouni, Nikos Koundouros, George Poulogiannis

**Affiliations:** a Signalling and Cancer Metabolism Team, Division of Cancer Biology, The Institute of Cancer Research, London, UK; b Division of Computational and Systems Medicine, Department of Surgery and Cancer, Imperial College, London, UK

**Keywords:** Cancer, nitrosative stress, PARK2, PTEN, S-nitrosylation

## Abstract

Cancer and Parkinson disease (PD) derive from distinct alterations in cellular processes, yet there are pathogenic mutations that are unequivocally linked to both diseases. Here we expand on our recent findings that loss of parkin RBR E3 ubiquitin protein ligase (*PRKN*, best known as *PARK2*)—which is genetically linked to PD—promotes cancer progression via redox-mediated inactivation of phosphatase and tensin homolog (PTEN) by S-nitrosylation.

Although cancer and Parkinson disease (PD) have opposing clinical manifestations, the observation that loss of parkin RBR E3 ubiquitin protein ligase (*PRKN*, best known as *PARK2*), also contributes to cancer progression suggests that additional common mechanisms may be involved in the progression of both diseases more so than previously appreciated. *PARK2* encodes the ubiquitin E3 ligase Parkin, and was first identified as a gene associated with the pathogenesis of early-onset PD,^1^ but was later linked with a wide range of disorders, most notably cancer, where it is a *bona fide* haploinsufficient tumor suppressor.^2^

In our recent study,^3^ we identified a novel role for the *PARK2* gene as a negative regulator of the phosphatidylinositol 3-kinase/AKT serine/threonine kinase 1 (PI3K/AKT) pathway, mediated by phosphatase and tensin homolog (PTEN). We showed that *PARK2* loss at both the DNA copy number and mRNA expression levels is observed on average in as high as one in three tumors and it correlates with poorer prognosis. Our findings demonstrated that *PARK2* depletion leads to a marked activation of the PI3K/AKT signaling, which is consistent with lower sensitivity to staurosporine-induced cell death and higher vulnerability to inhibitors of the PI3K/AKT pathway. In support of our data, Yeo and colleagues previously reported that ectopic PARK2 expression inhibits AKT activation, and mitigates cell proliferation and migration in glioma cells.^4^ Interestingly, we showed that the genetic background that faithfully recapitulates these phenotypes was wild-type for the tumor suppressor PTEN, suggesting that the functional contribution of *PARK2* depletion in the activation of PI3K-AKT pathway could be mediated, at least in part, by PTEN inactivation. Indeed, we showed that although *PARK2* depletion did not affect *PTEN* mRNA expression, it led to reduced PTEN protein levels and most importantly an ablation of its enzymatic activity, with a concomitant increase in phosphatidylinositol (3,4,5)-trisphosphate (PI(3,4,5)P_3_) and phosphatidylinositol (3,4)-bisphosphate (PtdIns(3,4)P_2_) levels.

In light of these data, along with previous compelling evidence that mitochondrial respiration defects lead to enhanced AKT activation through redox-mediated inactivation of PTEN,^5^ we studied the effect of *PARK2* depletion on cellular bioenergetics. Interestingly, *PARK2* knockdown cells showed a significant reduction in oxygen consumption rates, which was consistent with the activation of protein kinase AMP-activated catalytic subunit α 1 (AMPK) as the cellular energy sensor responding to adenosine triphosphate (ATP) depletion. *PARK2* knockdown also led to a major reduction in the anaplerotic substrates that contribute to nitric oxide (NO) synthesis, but with no significant difference in L-citrulline. These data, together with previous evidence that AMPK can directly phosphorylate nitric oxide synthase 3 (NOS3, best known as eNOS) at Ser1177 and Ser633^6^ enhancing NO bioavailability suggested that *PARK2* loss might play a prominent role in AMPK-mediated activation of eNOS. Indeed, we showed that *PARK2* depletion resulted in a marked increase in nitric oxide synthase (NOS) activity and the production of oxidized NO, leading to PTEN S-nitrosylation and its enhanced ubiquitin-proteasome degradation. Last but not least, *Park2* monoallelic or biallelic deletion in mice completely ablated Pten protein expression and was shown to promote tumor progression in *Pten* heterozygous knockout mice.

Our study highlights a critical role of nitrosative stress in regulating the activation of PI3K/AKT signaling (Fig. 1). Although PTEN appears critically important for this action, it is likely that there are other PTEN-independent mechanisms contributing to NO-induced activation of pro-survival signaling pathways. Notably, S-nitrosylation reactions, unlike in the case of PTEN, result in the activation of epidermal growth factor receptor (EGFR), proto-oncogene tyrosine-protein kinase Src,^7^ HRas proto-oncogene, GTPase (Ras)^8^ and can also modify hypoxia-inducible factor 1-α (HIF-1α),^9^ enhancing its accumulation and activity. Hence, *PARK2* loss could serve as a valuable biomarker for the identification of redox-dictated activation of oncogenic signaling pathways and ultimately guide the response to inhibitors of the aforementioned signaling pathways.

**Figure 1.**
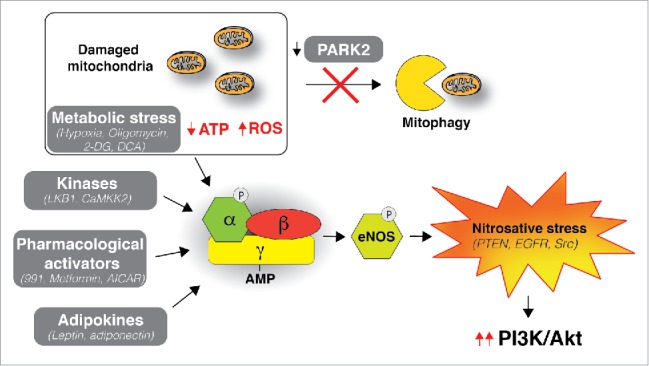
*PARK2* loss connects AMPK-mediated nitrosative stress to PI3K/AKT activation. Parkin RBR E3 ubiquitin protein ligase (PRKN, best known as PARK2) loss prevents degradation of damaged mitochondria by mitophagy leading to impaired respiration, lower adenosine triphosphate (ATP) levels, and an increase in ROS. Protein kinase AMP-activated catalytic subunit α 1 (AMPK) gets activated in response to metabolic stress and in turn induces activation of nitric oxide synthase 3 (NOS3, best known as eNOS), resulting in an increase in nitric oxide (NO) levels and concomitant inactivation of phosphatase and tensin homolog (PTEN) via S-nitrosylation, thereby promoting the activation of the phosphatidylinositol 3-kinase/AKT serine/threonine kinase 1 (PI3K/AKT) pathway. Other proteins in the pathway that could be the target of aberrant S-nitrosylation are EGFR and protooncogene tyrosine-protein kinase Src. AMPK can be activated by various additional modulators including (1) Metabolic stress that depletes ATP; e.g., by hypoxia, oligomycin, 2-Deoxy-glucose (2-DG) and dichloroacetate (DCA); (2) kinases; Serine/threonine-protein kinase STK11 (STK11, best known as LKB1) and Calcium/calmodulin-dependent protein kinase kinase 2 (CAMKK2); (3) Pharmacological activators; e.g., 991, Metfor-min and 5-Aminoimidazole-4-carboxamide ribonucleotide (AICAR); and (4) Adipokines; leptin and adiponectin. Metabolic stress-induced AMPK activation was shown to be sufficient to trigger PTEN S-nitrosylation even in the presence of functional PARK2.

In addition to the induction of NO signaling in the absence of functional PARK2, an important role for AMPK has also been defined. AMPK activation is stimulated in response to metabolic stress conditions, and we showed that it is sufficient to induce PTEN S-nitrosylation even in the presence of functional PARK2,^3^ demonstrating a novel functional crosstalk, whereby AMPK can contribute to AKT activation independent of its known role in the inhibition of the negative feedback loop mechanisms mediated by mechanistic target of rapamycin complex 1 (mTORC1) and ribosomal protein S6 kinase β-1 (S6K1). The role of AMPK, the central cellular metabolic sensor, in cancer is complex, and its potential as a therapeutic target is controversial. AMPK has been reported to act both as a tumor suppressor and oncogene based on the specific genetic, metabolic, and signaling contexts.^10^ Our results demonstrate that AMPK activation can confer the plasticity that cancer cells require to survive under conditions of metabolic stress through S-nitrosylation-mediated inhibition of PTEN (Fig. 1). Importantly, we found that cancer cells expressing the S-nitrosylation resistant isoform of PTEN (C83S) were significantly more sensitive to metabolic stress-inducing drugs 2-Deoxy-glucose (2-DG) and PDK inhibitor dichloroacetate (DCA),^3^ both of which result in the depletion of ATP and concomitant activation of AMPK. These findings are highly relevant in the context of the “biguanide paradox” theory proposed in the field based on reports that cancer cells are more susceptible to cell death by biguanides metformin, phenformin, or other compounds that cause metabolic stress [5-Aminoimidazole-4-carboxamide ribonucleotide (AICAR), salicylate, or 2-DG] when used in combination with agents that inhibit, rather than activate, AMPK.^10^

Although the precise implications of enhanced nitrosative stress and PI3K/AKT activation upon *PARK2* loss are likely to be different between cancer and PD, it is conceivable that they could promote or hinder cell survival in different disease settings. This is perhaps not surprising, when the post-mitotic nature of neuronal cells is considered; e.g. activation of the PI3K/AKT pathway may provide one means of promoting cell cycle re-entry in cancer cells, but could lead to the death of terminally differentiated neuronal cells. Moreover, cancer cells can exhibit higher detoxification capacity and/or adapt to oxidative and nitrosative stress-induced DNA damage, while considerable experimental evidence supports that the extensive production of reactive oxygen species (ROS) in the brain is the leading contributor to dopaminergic neuronal loss in PD.

Together, our data demonstrate that *PARK2* depletion contributes to PI3K/AKT activation via inactivation of PTEN by S-nitrosylation and provide several lines of evidence supporting the concept that inhibition of this pathway is important for PARK2's function as a tumor suppressor. It is anticipated that further studies identifying additional targets of S-nitrosylation might open up novel therapeutic opportunities for both cancer and PD.
